# How to build a device that cannot be built

**DOI:** 10.1007/s11128-015-1206-7

**Published:** 2015-12-28

**Authors:** Samuel J. Lomonaco

**Affiliations:** University of Maryland Baltimore County (UMBC), Baltimore, MD 21250 USA

**Keywords:** GHZ paradox, Quantum paradoxes, Quantum algorithms, Quantum computation, Quantum information, Quantum control, Distributed quantum algorithms, Quantum entanglement, Primary 68Q12, 81P68, 81P45, 81P40, 81P13, 81Q93, Secondary 03G12, 81P10

## Abstract

In this paper, we show how the GHZ paradox can be used to design a computing device that cannot be physically implemented within the context of classical physics, but nonetheless can be within quantum physics, i.e., in a quantum physics laboratory. This example gives an illustration of the many subtleties involved in the quantum control of distributed quantum systems. We also show how the second elementary symmetric Boolean function can be interpreted as a quantification of the nonlocality and indeterminism involved in the GHZ paradox.

## Introduction

This paper began with an invitation to give the Annual George Washington University Mathematics Department April Fools Day Lecture in April of 2014. After some thought, I decided what better topic to choose for the talk than how quantum mechanics makes fools of us all. For that reason, I chose to speak on the GHZ paradox, as embodied in Mermin’s machine [5].

In this paper, we show how the Greenberger–Horne–Zeilinger (GHZ) paradox can be used to design a computing device that cannot be physically implemented within the context of classical physics, but nonetheless can be within quantum physics, i.e., in a quantum physics laboratory. This example gives an illustration of the many subtleties involved in the quantum control of distributed quantum systems [8].

Corollary 1 in Sect. 6 can be interpreted as showing that the second elementary symmetric Boolean function $\sigma _{2}$ explicitly quantifies the nonlocality and indeterminism involved in the GHZ paradox.

## The device

**Fig. 1:**
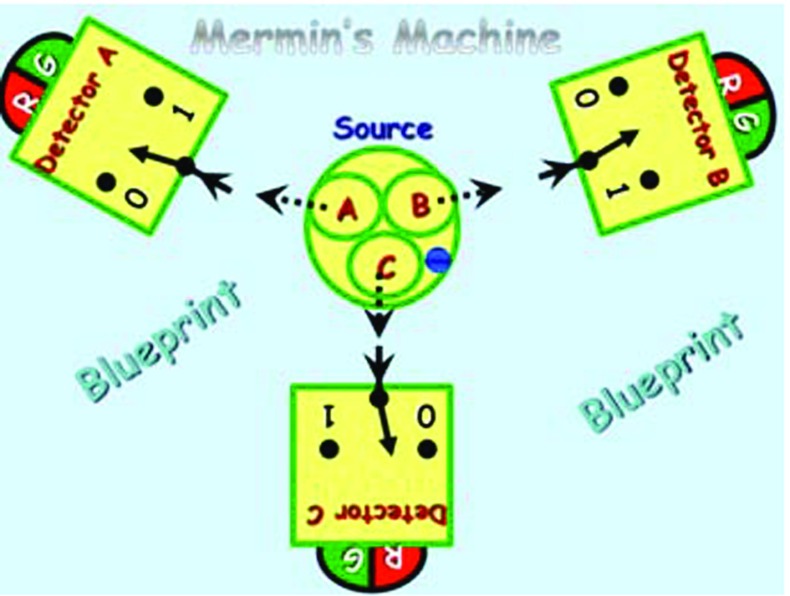
A blueprint of the device

A blueprint describing Mermin’s machine [5, 6] is shown below in Fig. 1:

**Fig. 2:**
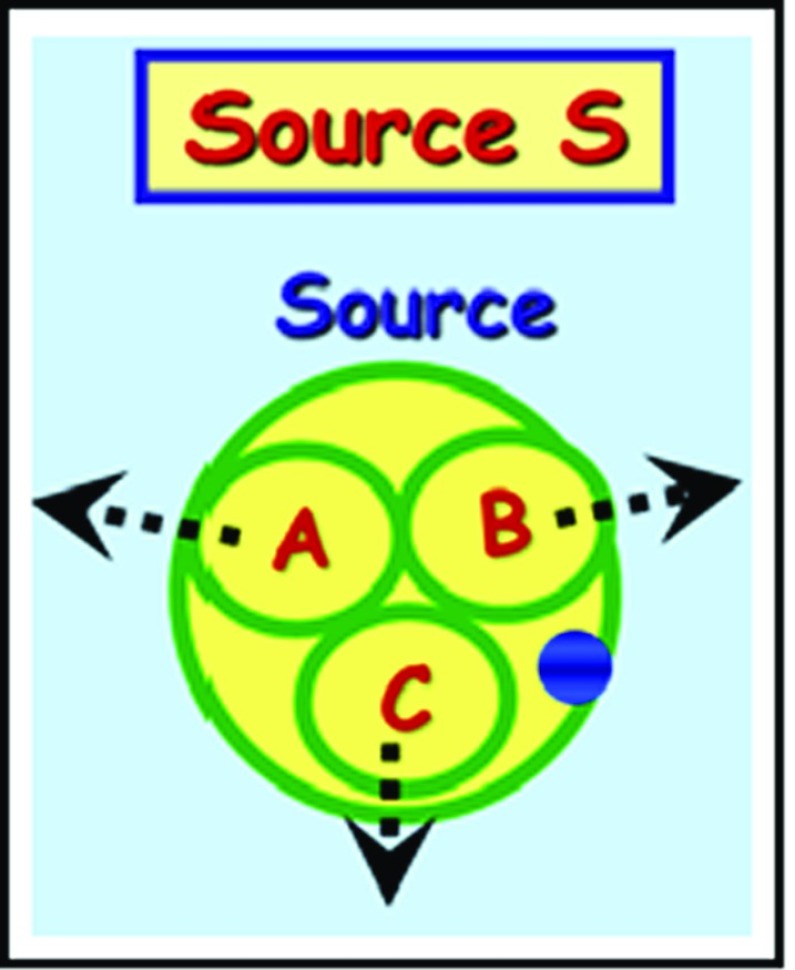
Source $$\varvec{S}$$

As illustrated, the device consists of two different types of components, i.e., a **source** $\varvec{S}$, and three identical **detectors**, labeled $\varvec{A}$, $\varvec{B}$, and $\varvec{C}$.

**Fig. 3:**
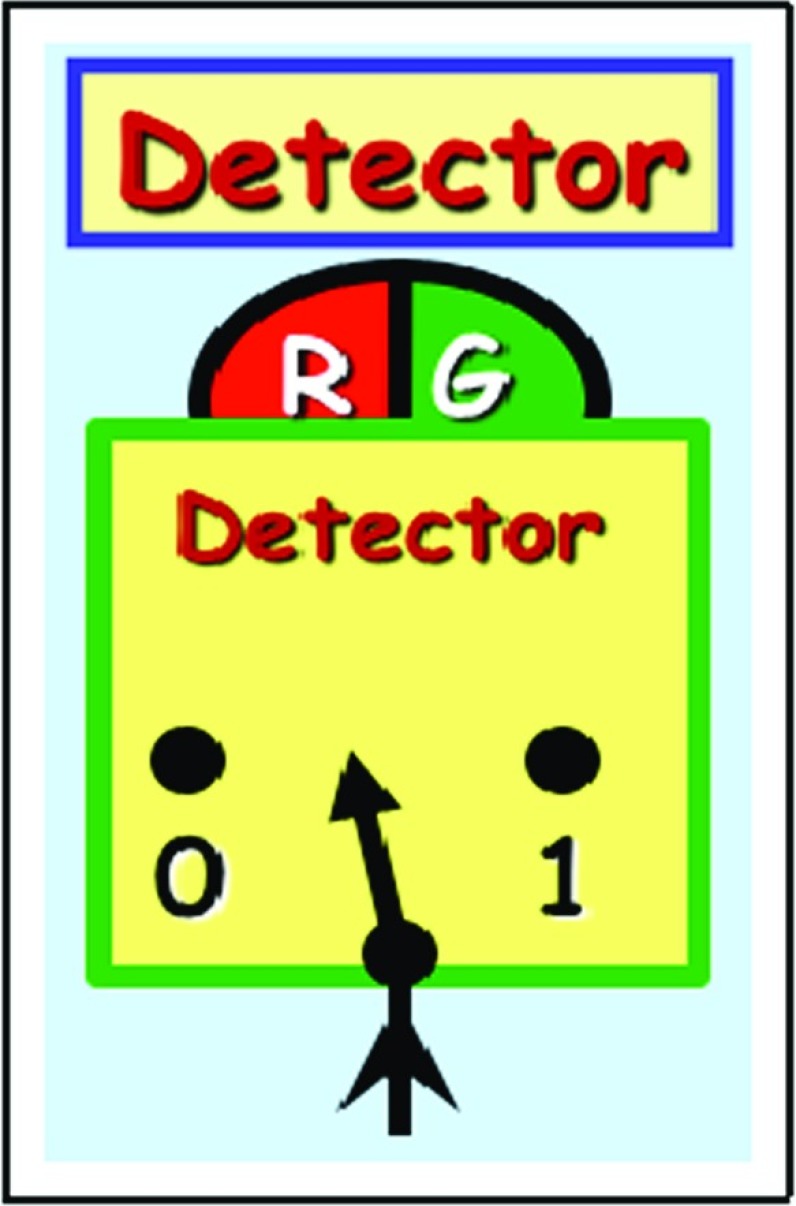
Detector $$\varvec{A}$$, $$\varvec{B}$$, or $$\varvec{C}$$

The source, as illustrated below in Fig. 2, is a device that contains three objects, called **particles**, labeled $\varvec{A}$, $\varvec{B}$, and $\varvec{C}$, and a **blue button**, which, when pressed, ejects the three particles $\varvec{A}$, $\varvec{B}$, and $\varvec{C}$ in the directions toward the detectors $\varvec{A}$, $\varvec{B}$, and $\varvec{C}$, respectively (Fig. 3).

Each detector, upon encountering an incoming particle, flashes either red $\varvec{R}$ or green $\varvec{G}$. Moreover, each detector has a switch with two settings $\varvec{0}$ and $\varvec{1}$, which is randomly set at anytime before the arrival of the particle.

As stated below in the design specifications, the only switch settings of interest are those for which an odd number of the three switches is set to $\varvec{1}$, i.e.,No other switch settings are important, i.e., of interest.

The design specifications are as follows:

*Spec 1*: After all particles are detected, for switch settings $\varvec{001}$, $\varvec{010}$, and $\varvec{100}$, **only an odd number** of the detectors flash red $\varvec{R}$.
*Spec 2*: After all particles are detected, for switch setting $\varvec{111}$, **only an even number** of detectors flash red $\varvec{R}$.

These design specifications are subject to the following three constraints:

*Constraint 1*: The detectors cannot communicate with one another. (They are separated by a spacelike distance.)
*Constraint 2*: After being ejected from the source *S*, the particles can no longer communicate with one another.
*Constraint 3*: The particles only communicate with the detector upon impact.

## It can’t be built!

Because of the above constraints, each particle must locally carry instructions telling its respective detector whether to flash red $\varvec{R}$ or green $\varvec{G}$.

For example, particle $\varvec{A}$ must carry a **local instruction** $f_{A}\left( s_{A}\right)$ of the formwhere $c_{A0}=\varvec{R}$ or $\varvec{G}$ and $c_{A1}=\varvec{R}$ or $\varvec{G}$ for switch settings $s_{A}=0$ or 1, respectively. In like manner, the remaining two particles $\varvec{B}$ and $\varvec{C}$ must carry local instructions $f_{B}\left( s_{B}\right)$ and $f_{C}\left( s_{C}\right)$, respectively.

Let us rename the colors $\varvec{R}$ and $\mathbf {G}$ as $\mathbf {R}=1$ and $\mathbf {G}=0$, respectively. Thus, for each $j=A,B,C$, the local instruction $f_{j}\left( s_{j}\right)$ is simply a **Boolean function**

$$\begin{aligned} f_{j}:\left\{ 0,1\right\} \longrightarrow \left\{ 0,1\right\} \text { .} \end{aligned}$$

It is now immediate that Specs 1 and 2 are equivalent to the following linear system of equations:

$$\begin{aligned} \left\{ \begin{array}{l} f_{A}(0)+f_{B}(0)+f_{C}(1)=1\left( {\text {mod}}2\right) \\ f_{A}(0)+f_{B}(1)+f_{C}(0)=1\left( {\text {mod}}2\right) \\ f_{A}(1)+f_{B}(0)+f_{C}(0)=1\left( {\text {mod}}2\right) \\ f_{A}(1)+f_{B}(1)+f_{C}(1)=0\left( {\text {mod}}2\right) \end{array} \right. \end{aligned}$$

which is obviously inconsistent.

In other words, the device cannot be built! It’s simply impossible. $\square$

## Oh, but it can be built!

However, within the context of quantum physics, it can actually be built, i.e., can be physically implemented.

But before we can show how this device can actually be built, we need a few definitions.

### **Definition 1**

We define a **Boolean unitary transformation** as a map from $\left\{ 0,1\right\} ^{k}$ into a group of unitary transformations. In like manner, a **Boolean Hermitian operator** is defined as a map from $\left\{ 0,1\right\} ^{k}$ into an algebra of observables. If *b* ($=0$ or 1) and if *U* is a unitary transformation, then $U^{b}$ will denote the Boolean unitary transformationwhere *I* denotes the identity operator. In like manner, if $\varOmega$ is an observable, then $b\varOmega$ will denote the Boolean observablewhere *O* denotes the zero operator.

### *Remark 1*

In other words, Boolean unitary and Boolean Hermitian operators are unitary and Hermitian transformations controlled by classical bits.

### *Remark 2*

There is much more that could be said in regard to Boolean unitary and Hermitian operators. But that would take us too far afield of the intended objectives of this paper. So the following will have to suffice: Let $\mathbb {B}$ be a Boolean algebra or Boolean ring. Let $\mathbb {U}$ be a unitary group, and let $\mathbf {u}$ denote its Lie algebra. The set $\mathbb {U}^{\mathbb {B}}=map\left( \mathbb {B},\mathbb {U}\right)$ of Boolean unitary operators forms a Lie group containing the group $\mathbb {U}$ as a sub-Lie group. Moreover, the set $\mathbf {u}^{\mathbb {B}}=map\left( \mathbb {B},\mathbf {u}\right)$ of Boolean Skew Hermitian operators is the Lie algebra of $\mathbb {U}^{\mathbb {B}}$ and contains $\mathbf {u}$ as a sub-Lie algebra.

Let *X*, *Y*, *Z*, respectively, denote the Pauli spin operators

$$\begin{aligned} X=\left( \begin{array} [c]{rr} 0 &{} 1\\ 1 &{} 0 \end{array} \right) \text {,} \ Y=\left( \begin{array} [c]{rr} 0 &{} -i\\ i &{} 0 \end{array} \right) \text {,} \ Z=\left( \begin{array} [c]{rr} 1 &{} 0\\ 0 &{} -1 \end{array} \right) . \end{aligned}$$

Moreover, let *H* denote the Hadamard gate

$$\begin{aligned} H=\frac{1}{\sqrt{2}}\left( \begin{array} [c]{rr} 1 &{} 1\\ 1 &{} -1 \end{array} \right) \text { ,} \end{aligned}$$

and let *U* be the single-qubit gate

$$\begin{aligned} U=e^{\left[ \frac{i\pi }{3}\left( \frac{X+Y+Z}{\sqrt{3}}\right) \right] }=\frac{1+i}{2}\left( \begin{array} [c]{rr} 1 &{} 1\\ i &{} -i \end{array} \right) \text { .} \end{aligned}$$

**Fig. 4:**
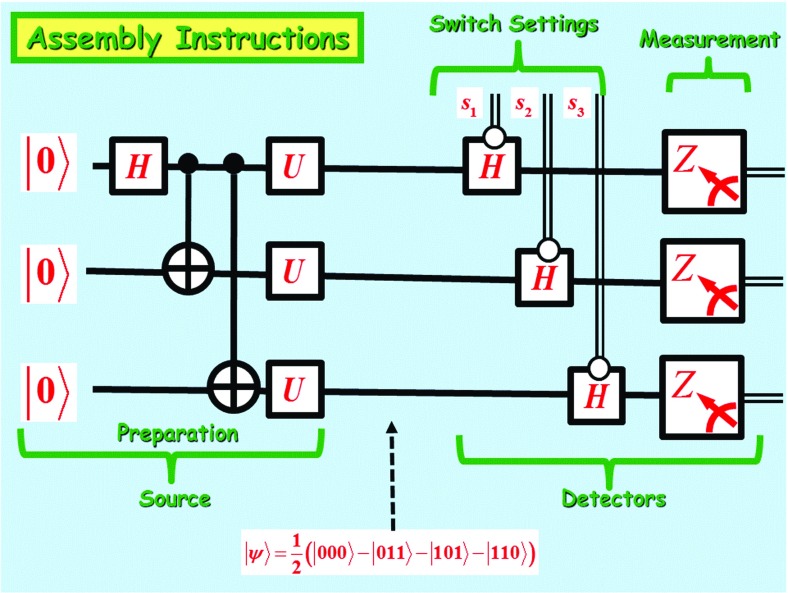
Wiring diagram of device

A wiring diagram summarizing a physical implementation of Mermin’s machine is shown in Fig. 4.

In this diagram, a single line indicates a wire carrying a qubit and a double line indicates a wire carrying a classical bit. The graphicsdenote, respectively, a **Controlled-Not** and a **measurement in the standard basis.** Finally the graphic

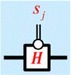



denotes the Boolean gate $H^{s_{j}^{*}}$, controlled by the classical bit $s_{j}^{*}$, where $s_{j}^{*}$ denotes the **complement** of the *j*-th switch setting $s_{j}$. In other words,


### *Remark 3*

**Fig. 5:**
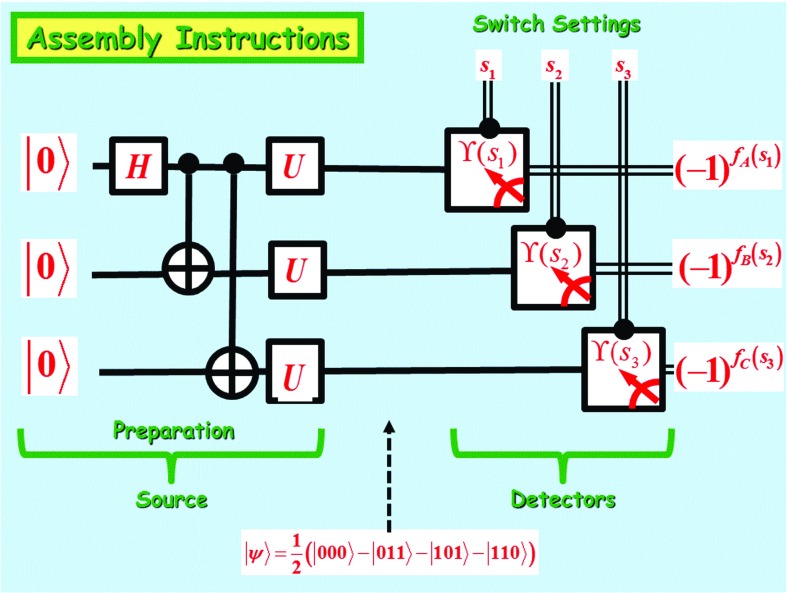
An equivalent wiring diagram for Mermin’s machine, where $\Upsilon \left( s_{j}\right)$ is the Boolean observable $\Upsilon \left( s_{j}\right) =s_{j}^{*}X+s_{j}Z$

Please note that $HZH=X$. Hence, if $\left| \varphi \right\rangle$ is a single-qubit state, then measurement of $H^{s_{j}^{*}}\left| \varphi \right\rangle$ with respect to the observable *Z* is equivalent to measurement of $\left| \varphi \right\rangle$ with respect to the Boolean observable $H^{s_{j}^{*}}ZH^{s_{j}^{*}}=s_{j}^{*}X+s_{j}Z$. So, each detector portion of the wiring diagram can be simplified to a local measurement with respect to the Boolean observable $s_{j}^{*}X+s_{j}Z$, for $j=1,2,3$ (please refer to Fig. 5). In fact, each detector portion of the diagram can be even further simplified to local measurement of the GHZ state with respect to Boolean observable $U^{\dag }\left( s_{1}^{*} X+s_{1}Z\right) U=s_{j}^{*}Y+s_{j}X$, for $j=1,2,3$.

The three leftmost gates provide a preparation of the GHZ state

$$\begin{aligned} \frac{1}{\sqrt{2}}\left( \left| 000\right\rangle +\left| 111\right\rangle \right) \text { .} \end{aligned}$$

The local unitary^1^ transformation $U^{\otimes 3}=U\otimes U\otimes U$ transforms the GHZ state into the entangled state

$$\begin{aligned} \left| \psi \right\rangle =\frac{1}{2}\left( \left| 000\right\rangle -\left| 011\right\rangle -\left| 101\right\rangle -\left| 110\right\rangle \right) \text { ,} \end{aligned}$$

which will be used to control the flashing light patterns of the three detectors.^2^

Let $\mathcal {H}_{even}$ and $\mathcal {H}_{odd}$ denote the Hilbert subspaces of the underlying three-qubit Hilbert space $\mathcal {H}$ spanned, respectively, by the standard basis elements labeled by bit strings of even and odd Hamming weight. It now follows from the following table:that

$$\begin{aligned} \left( H^{s_{1}^{*}}\otimes H^{s_{2}^{*}}\otimes H^{s_{3}^{*} }\right) \left| \psi \right\rangle \in \left\{ \begin{array} [c]{ll} \mathcal {H}_{even} &{} \hbox {if} \,s=111\\ &{} \\ \mathcal {H}_{odd} &{} \hbox {if} \, s=001,010,100 \end{array} \ \ \ \right. \end{aligned}$$

Thus, if the switch setting is $s=111$, application of each and all local detector measurements with respect to the standard basis (no matter in which temporal order) will project the state $\left( H^{s_{1}^{*}}\otimes H^{s_{2}^{*}}\otimes H^{s_{3}^{*}}\right) \left| \psi \right\rangle$ into $\mathcal {H}_{even}$, necessarily resulting in a standard basis state $\left| c_{1}c_{2}c_{3}\right\rangle$ of even Hamming weight, and corresponding eigenvalues $(-1)^{c_{1}},(-1)^{c_{2}},(-1)^{c_{3}}$ with $c_{1}+c_{2}+c_{3}=0({\text {mod}}2)$. Using the same argument for the switch settings $s=001,010,001$, the three local detector measurements of $\left( H^{s_{1}^{*}}\otimes H^{s_{2}^{*}}\otimes H^{s_{3}^{*} }\right) \left| \psi \right\rangle$ will result in a standard basis element $\left| c_{1}c_{2}c_{3}\right\rangle$ of odd Hamming weight with corresponding eigenvalues $(-1)^{c_{1}},(-1)^{c_{2}},(-1)^{c_{3}}$ with $c_{1}+c_{2}+c_{3}=1({\text {mod}}2)$.

Thus, using $c_{j}=0$ as the control bit instruction to flash Green G and $c_{j}=1$ as the control bit instruction to flash Red, we have shown that the device defined by the wiring diagram satisfies all the required specs and constraints.

So the device can be built after all!

## Why?

So where has the impossibility argument given in Sect. 3 of this paper gone awry?

Certainly the proof in Sect. 3 of this paper of the following proposition, on which the proof of impossibility is based, is beyond reproach:

### **Proposition 1**

There exist no Boolean functions

$$\begin{aligned} f_{A}:\left\{ 0,1\right\} \longrightarrow \left\{ 0,1\right\} ,\quad f_{B}:\left\{ 0,1\right\} \longrightarrow \left\{ 0,1\right\} ,\quad f_{C}:\left\{ 0,1\right\} \longrightarrow \left\{ 0,1\right\} \end{aligned}$$

such that

$$\begin{aligned} f_{A}\left( s_{1}\right) +f_{B}\left( s_{2}\right) +f_{C}\left( s_{3}\right) \equiv \left\{ \begin{array} [c]{ll} 1\ \left( {\text {mod}}2\right) &{} \text {if }s=\left( s_{1},s_{2} ,s_{3}\right) =001\text {, }010\text {, or }100\text {.}\\ 0\ \left( {\text {mod}}2\right) &{} \text {if }s=\left( s_{1},s_{2} ,s_{3}\right) =111 \end{array} \right. \end{aligned}$$

The logic is flawless.^3^ But the crux of the matter is that the argument of impossibility found in Sect. 3 is only as sound as the assumptions upon which it is based.

More specifically, the argument of impossibility fails because at least one of the following two tacitly assumed premises is false:

Premise 1. **Reality Principle:** *What is measured is completely determined before it is measured* (for a more refined definition of this principle and the concept of an element of reality, please refer to [1] and [7]).

Premise 2. **Principle of Locality:** *Spacelike separated regions of spacetime are physically independent*.

### *Remark 4*

It is not clear that these are fully independent principles. For how can that which is not fully determined already be localized? Moreover, can that which is not localized already be fully determined?

The above two premises lead to the following unfounded conclusions:

Unfounded Conclusion 1. *Based on Premise 1 (The Reality Principle), the detector lamp instructions* $$f_{A}$$, $$f_{B} $$, $$f_{C}$$ *must already be predetermined well-defined total functions* 4 at the time of particle ejection.

Unfounded Conclusion 2. *Based on Premise 2 (The Principle of Locality), the detector lamp instructions* $f_{A}$ *,* $f_{B}$ *,* $f_{C}$ *must be local. Hence,* $f_{j}$ *is a function only of the* *j* *th switch setting* $s_{j}$ *and independent of the two other switch settings.*

We will show in the next section that the detector lamp instructions $f_{A}$, $f_{B}$, $f_{C}$ are neither predetermined well-defined functions before ejection, nor local independent functions.

## Under the mathematical microscope

It is instructive to take a closer look at Mermin’s machine.

We will now explicitly compute the random functions $f_{A}$, $f_{B}$, $f_{C}$. In so doing, we will find, contrary to the unfounded conclusions given in the previous section, that these functions are:

1. Random partial functions,
2. Global interdependent functions of the switch settings, and
3. Not fully defined until measured by the detectors.

For reasons of transparency, it will prove more convenient to work with the equivalent wiring diagram shown in Fig. 5, where


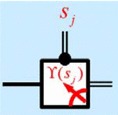


denotes the $s_{j}$-controlled gate for the Boolean observableThat this wiring diagram is equivalent to the one found in Fig. 4 follows from the fact that $HZH=X$. Hence, measurement of $H^{s_{j}^{*}}\left| \psi \right\rangle$ with respect to *Z* is equivalent to measurement of $\left| \psi \right\rangle$ with respect to $H^{s_{j}^{*}} ZH^{s_{j}^{*}}=s_{j}^{*}X+s_{j}Z$.

We will also need to use the **quantum measurement function** *Q*, which takes as input a pair consisting of an existing quantum state and a quantum observable and then upon evaluation produces as output a pair consisting of a resulting eigenstate and the corresponding eigenvalue. For example, if $\rho$ is a density operator representing the state of a quantum system and if $\varOmega$ an observable with spectral decomposition

$$\begin{aligned} \varOmega ={\displaystyle \sum \limits _{j}^{n}} \lambda _{j}P_{j}\text {,} \end{aligned}$$

then on evaluation $Q\left( \rho ,\varOmega \right)$ produces

$$\begin{aligned} Q\left( \rho ,\varOmega \right) =\left( \frac{P_{j}\rho P_{j}}{Tr\left( P_{j}\rho \right) },\lambda _{j}\right) \text { , } \end{aligned}$$

where $P_{j}$ is the projection operator for the eigenspace corresponding to the eigenvalue $\lambda _{j}$.

Please note that the function *Q* is a random output function, very much like the random number generator found on most classical computers, except that its output is not pseudorandom, but actually truly random. A pseudorandom number generator is a **predeterministic** function, i.e., a function fully predefined before evaluation, which upon evaluation deterministically produces an output. On the other hand, the function *Q* is **indeterministic**,^5^ i.e., it is a function that is not fully defined (and not fully determined) as a function until it is evaluated.

We finally are ready to take a closer look at the implementation of Mermin’s machine, as described by the wiring diagram found in Fig. 5.

After the state preparation of the entangled state $\left| \psi \right\rangle$ and before ejection of the particles, the detector lamp instructions $f_{A}$, $f_{B}$, $f_{C}$ are indeterministic, i.e., only partially defined (and only partially localized) by the entangled state $\left| \psi \right\rangle$. This is a result of the state of each individual qubit of $\left| \psi \right\rangle$ being indeterministic, i.e., not yet fully defined, and not yet fully localized.

In Sect. 4, it was pointed out that the property that the final resulting light pattern always satisfies the machine specifications and constraints is independent of the temporal order of the detector measurements. For this reason, we focus only on the case for which the detector measurements occur in the temporal order $t_{A}<t_{B}<t_{C}$, where $t_{A}$, $t_{B}$, $t_{C}$ denote the measurement times for detectors *A*, *B*, *C*, respectively.

### *Remark 5*

The topic of the temporal order of measurements is remarkably subtle. To say that the detector light pattern is independent of the order of the measurements is counterfactual and hence physically meaningless. However, it is meaningful (not counterfactual) to say that the state specifications and constraints are met, independent of the order of measurements. On the other hand, because of relativity, there can be, for each possible temporal order, a different observer that observes the measurements in that order. The fact that each of three different observers sees the measurements in a different temporal order is not counterfactual because all observers are viewing the same measurements.

We recall that the spectral decompositions of the Pauli spin operators *X* and *Z* are, respectively,

$$\begin{aligned} X=\left( -1\right) ^{0}P_{+}+\left( -1\right) ^{1}P_{-} \quad \text {and} \quad Z=\left( -1\right) ^{0}P_{0}+\left( -1\right) ^{1}P_{1}\text { ,} \end{aligned}$$

where

$$\begin{aligned} \left\{ \begin{array} [c]{c} P_{+}=\left| +\right\rangle \left\langle +\right| \\ \\ P_{-}=\left| -\right\rangle \left\langle -\right| \end{array} \right. \quad \text {and} \quad \left\{ \begin{array} [c]{c} P_{0}=\left| 0\right\rangle \left\langle 0\right| \\ \\ P_{1}=\left| 1\right\rangle \left\langle 1\right| \end{array} \right. \text { ,} \end{aligned}$$

and where

$$\begin{aligned} \left\{ \begin{array} [c]{c} \left| +\right\rangle =\frac{\left| 0\right\rangle +\left| 1\right\rangle }{\sqrt{2}}\\ \\ \left| -\right\rangle =\frac{\left| 0\right\rangle -\left| 1\right\rangle }{\sqrt{2}} \end{array} \right. \text { .} \end{aligned}$$

### **Notation 1**

In the calculations to follow, we use the following notational convention:



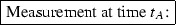


At the time $t_{A}$, the function $f_{A}\left( s_{1}\right)$ is evaluated as follows:

$$\begin{aligned}&Q\left( Tr_{23}\left( \left| \psi \right\rangle \left\langle \psi \right| \right) ,s_{1}^{*}X+s_{1}Z\right) \\&\quad =\left( \frac{P_{j_{1}^{s_{1}^{*}}}Tr_{23}\left( \left| \psi \right\rangle \left\langle \psi \right| \right) P_{j_{1}^{s_{1}^{*}}}}{Tr\left( P_{j_{1}^{s_{1}^{*}}}Tr_{23}\left( \left| \psi \right\rangle \left\langle \psi \right| \right) \right) },\left( -1\right) ^{j_{1} }\right) \Longrightarrow f_{A}(s_{1})=j_{1}\text { ,} \end{aligned}$$

where $j_{1}=0$ or 1, and where $Tr_{23}\left( \left| \psi \right\rangle \left\langle \psi \right| \right)$ is the partial trace of $\left| \psi \right\rangle \left\langle \psi \right|$ over qubits 2 and 3. The resulting state of the three qubits is

$$\begin{aligned} \left| \psi ^{\prime }\right\rangle =\frac{\left( P_{j_{1}^{s_{1}^{*}} }\otimes 1\otimes 1\right) \left| \psi \right\rangle }{\sqrt{\left\langle \psi \left| P_{j_{1}^{s_{1}^{*}}}\otimes 1\otimes 1\right| \psi \right\rangle }}\text { .} \end{aligned}$$

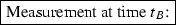


At the time $t_{B}$, the function $f_{B}\left( s_{2}\right)$ is evaluated as follows:

$$\begin{aligned}&Q\left( Tr_{13}\left( \left| \psi ^{\prime }\right\rangle \left\langle \psi ^{\prime }\right| \right) ,s_{2}^{*}X+s_{2}Z\right) \\&\quad =\left( \frac{P_{j_{2}^{s_{2}^{*}}}Tr_{13}\left( \left| \psi ^{\prime }\right\rangle \left\langle \psi ^{\prime }\right| \right) P_{j_{2} ^{s_{2}^{*}}}}{Tr\left( P_{j_{2}^{s_{2}^{*}}}Tr_{13}\left( \left| \psi ^{\prime }\right\rangle \left\langle \psi ^{\prime }\right| \right) \right) },\left( -1\right) ^{j_{2}}\right) \Longrightarrow f_{A} (s_{2})=j_{2}\text { , } \end{aligned}$$

where $j_{2}=0$ or 1, and where $Tr_{13}\left( \left| \psi ^{\prime }\right\rangle \left\langle \psi ^{\prime }\right| \right)$ is the partial trace of $\left| \psi ^{\prime }\right\rangle \left\langle \psi ^{\prime }\right|$ over qubits 1 and 3. The resulting state of the three qubits is

$$\begin{aligned} \left| \psi ^{\prime \prime }\right\rangle =\frac{\left( 1\otimes P_{j_{2}^{s_{2}^{*}}}\otimes 1\right) \left| \psi ^{\prime }\right\rangle }{\sqrt{\left\langle \psi \right| \left( 1\otimes P_{j_{2}^{s_{2}^{*}} }\otimes 1\right) \left| \psi ^{\prime }\right\rangle }}\text { .} \end{aligned}$$

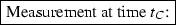


At the time $t_{C}$, the function $f_{C}\left( s_{3}\right)$ is evaluated as follows:

$$\begin{aligned}&Q\left( Tr_{12}\left( \left| \psi ^{\prime \prime }\right\rangle \left\langle \psi ^{\prime \prime }\right| \right) ,s_{3}^{*} X+s_{3}Z\right) \\&\quad =\left( \frac{P_{j_{3}^{s_{3}^{*}}}Tr_{12}\left( \left| \psi ^{\prime \prime }\right\rangle \left\langle \psi ^{\prime \prime }\right| \right) P_{j_{3}^{s_{3}^{*}}}}{Tr\left( P_{j_{3} ^{s_{3}^{*}}}Tr_{12}\left( \left| \psi ^{\prime \prime }\right\rangle \left\langle \psi ^{\prime \prime }\right| \right) \right) },\left( -1\right) ^{j_{3}}\right) \Longrightarrow f_{A}(s_{3})=j_{3}\text { , } \end{aligned}$$

where $j_{3}=0$ or 1, and where $Tr_{12}\left( \left| \psi ^{\prime \prime }\right\rangle \left\langle \psi ^{\prime \prime }\right| \right)$ is the partial trace of $\left| \psi ^{\prime \prime }\right\rangle \left\langle \psi ^{\prime \prime }\right|$ over qubits 1 and 2 The resulting state of the three qubits is

$$\begin{aligned} \left| \psi ^{\prime \prime \prime }\right\rangle =\frac{\left( 1\otimes 1\otimes P_{j_{3}^{s_{3}^{*}}}\right) \left| \psi ^{\prime \prime }\right\rangle }{\sqrt{\left\langle \psi ^{\prime \prime }\right| \left( 1\otimes 1\otimes P_{j_{3}^{s_{3}^{*}}}\right) \left| \psi ^{\prime \prime }\right\rangle }}\text { .} \end{aligned}$$

### *Remark 6*

Please note that each of the instructions $f_{A}\left( s\right)$, $f_{B}\left( s\right)$, $f_{C}\left( s\right)$ can only be a nonlocal function of $s=\left( s_{1},s_{2},s_{3}\right)$. For from relativity, there can be three different observers Alice, Bob, and Charlie each observing the same measurements, but each observing the same measurements in the three different temporal orders $t_{A}<t_{B}<t_{C}$, $t_{B}<t_{C}<t_{A}$, $t_{C}<t_{A}<t_{B}$, respectively. If Alice observes $f_{A}$ as only a function of $s_{1}$, so would Bob and Charlie.

We are now in a position to explicitly quantify the interdependence of the random Boolean partial functions $f_{A}$, $f_{B}$, $f_{C}$. To do so, we will make use of the following well-known combinatorial formula [3]:

### **Theorem 1**

Let $b=\left( b_{1},b_{2},b_{3},\ldots ,b_{n}\right)$ be a binary string of length $n>0$. The binary expansion of the Hamming weight $Wt\left( b\right)$ of *b* is given by the following formula:

$$\begin{aligned} Wt\left( b\right) = {\displaystyle \sum \limits _{k=0}^{O\left( \log n\right) }} \sigma _{2^{k}}\left( b\right) \cdot 2^{k}\text { ,} \end{aligned}$$

where $\sigma _{2^{k}}\left( b\right)$ denotes the $2^{k}$-th elementary symmetric function modulo 2, i.e.,

$$\begin{aligned} \sigma _{2^{k}}\left( b\right) = {\displaystyle \sum \limits _{1\le \ell _{1}<\ell _{2}<\ldots <\ell _{n}\le 2^{k}}} b_{\ell _{1}}b_{\ell _{2}}b_{\ell _{3}}\cdots b_{\ell _{n}}\quad \left( {\text {mod}}2\right) \text { .} \end{aligned}$$

In light of the above theorem, an immediate consequence of the above measurement calculations is the following lemma and corollary:

### **Lemma 1**

If the switch setting $s=\left( s_{1},s_{2},s_{3}\right)$ is of odd Hamming weight, then

$$\begin{aligned} \left( P_{j_{1}^{s_{1}^{*}}}\otimes P_{j_{2}^{s_{2}^{*}}} \otimes 1\right) \left| \psi \right\rangle \text { lies in }\left( 1\otimes 1\otimes P_{j_{3}^{s_{3}^{*}}}\right) \mathcal {H}\text { ,} \end{aligned}$$

where

$$\begin{aligned} j_{3}=j_{1}+j_{2}+\sigma _{2}\left( s\right) +1\quad \left( {\text {mod}}2\right) \text { ,} \end{aligned}$$

and where $\sigma _{2}\left( s\right)$ denotes the second elementary symmetric function

$$\begin{aligned} \sigma _{2}\left( s\right) =s_{1}s_{2}+s_{2}s_{3}+s_{3}s_{1}\text { .} \end{aligned}$$

Thus,

$$\begin{aligned} \left| \psi ^{\prime \prime \prime }\right\rangle =\left| \psi ^{\prime \prime }\right\rangle \text { .} \end{aligned}$$

### **Corollary 1**

For a switch setting $s=\left( s_{1},s_{2},s_{3}\right)$ of odd Hamming weight, the detector lamp instructions $f_{A}$, $f_{B}$, $f_{C}$ are the random partial functions given by:

$$\begin{aligned} \left\{ \begin{array} [c]{l} f_{A}\left( s\right) =j_{1}\\ \\ f_{B}\left( s\right) =j_{2}\\ \\ f_{C}\left( s\right) =j_{3} \end{array} \right. \text { ,} \end{aligned}$$

with the Boolean algebraic dependence

$$\begin{aligned} f_{A}\left( s\right) +f_{B}\left( s\right) +f_{C}\left( s\right) =\sigma _{2}\left( s_{1},s_{2},s_{3}\right) +1\quad \left( {\text {mod}} 2\right) \text { ,} \end{aligned}$$

where $\sigma _{2}$ denotes the second elementary symmetric function

$$\begin{aligned} \sigma _{2}\left( s_{1},s_{2},s_{3}\right) =s_{1}s_{2}+s_{2}s_{3}+s_{3} s_{1}\text { .} \end{aligned}$$

Hence, the random Boolean instruction functions $f_{A}$, $f_{B}$, $f_{C}$ are global and interdependent partial functions, thereby refuting Unfounded Conclusions 1 and 2, found in Sect. 5 of this paper.

### *Remark 7*

It is interesting to note that the Boolean function $\sigma _{2}\left( s_{1},s_{2},s_{3}\right)$, involved in the above algebraic interdependence, in some way fully encapsulates the entire paradox. In other words, this second elementary symmetric Boolean function somehow quantifies the nonlocality and the indeterminism involved in the GHZ paradox.

## Conclusion?

We conclude with no conclusion, but with a question:

### **Question 1**

Is quantum mechanics trying to tell us that the very fabric of reality is indeterminate, i.e., not fully defined until it is observed?

## Footnotes

1 It is important to note that $U^{\otimes 3}$ is a local unitary transformation that does not change entanglement type. For a better understanding of the significance of this fact, please refer to [4].

2 For a more in-depth explanation of the use of entanglement as a distributed control mechanism, please refer to [8].

3 Actually, as we will see, the above proposition is a proof of the counterfactuality of the lamp instructions being total functions, and not a proof that the device cannot be built.

4 A **total function** is a function that is defined for all possible values of its arguments. A **partial function** is a function defined for some of its argument values, but not necessarily all. For more information, please refer to any text on recursive function theory.

5 Please note that we have avoided use of the term “nondeterministic” because this term has an entirely different meaning in the theory of computation.
